# Network Pharmacology Analysis of* Damnacanthus indicus C.F.Gaertn* in Gene-Phenotype

**DOI:** 10.1155/2019/1368371

**Published:** 2019-02-17

**Authors:** Shengrong Long, Caihong Yuan, Yue Wang, Jie Zhang, Guangyu Li

**Affiliations:** ^1^Department of Neurosurgery, The First Affiliated Hospital of China Medical University, NanJing Bei Road, Heping District, Shenyang, 110001, LiaoNing Province, China; ^2^Department of Chinese Medicine, The First Affiliated Hospital of China Medical University, NanJing Bei Road, Heping District, Shenyang, 110001, LiaoNing Province, China

## Abstract

*Damnacanthus indicus C.F.Gaertn* is known as Huci in traditional Chinese medicine. It contains a component having anthraquinone-like structure which is a part of the many used anticancer drugs. This study was to collect the evidence of disease-modulatory activities of Huci by analyzing the published literature on the chemicals and drugs. A list of its compounds and direct protein targets is predicted by using Bioinformatics Analysis Tool for Molecular Mechanism of TCM. A protein-protein interaction network using links between its directed targets and the other known targets was constructed. The DPT-associated genes in net were scrutinized by WebGestalt. Exploring the cancer genomics data related to Huci through cBio Portal. Survival analysis for the overlap genes is done by using UALCAN. We got 16 compounds and it predicts 62 direct protein targets and 100 DPTs and they were identified for these compounds. DPT-associated genes were analyzed by WebGestalt. Through the enrichment analysis, we got top 10 identified KEGG pathways. Refined analysis of KEGG pathways showed that one of these ten pathways is linked to Rap1 signaling pathway and another one is related to breast cancer. The survival analysis for the overlap genes shows the significant negative effect of these genes on the breast cancer patients. Through the research results of* Damnacanthus indicus C.F.Gaertn*, it is shown that medicine network pharmacology may be regarded as a new paradigm for guiding the future studies of the traditional Chinese medicine in different fields.

## 1. Introduction

 Anthraquinones are widely found in many plants such as Rubiaceae, Polygonaceae, and Legumes. A lot of studies have previously reported its extraction and separation, composition analysis, and pharmacological effects. The doxorubicin and mitoxantrone are the commonly used anticancer drugs, whose parent nucleus is anthraquinones [1]. However, the mechanism of antitumor action of anthraquinones is not fully understood yet.* Damnacanthus indicus C.F.Gaertn*,* Damnacanthus giganteus, rubia cordifolia L, *and* prismatomeris tetrandra* are widely used traditional anticancer herbs. Damnacanthal (1-methoxy-2-aldehyde-3-hydroxyanthraquinone) is an important secondary metabolite found in these herbs [2–4]. Many studies have shown that this compound exerts the cytotoxic effects [5] by inhibiting the cell proliferation [6, 7] and by inducing apoptosis of cancer cells [7–9]. With the development of human genomics, some new methods have been proposed for the discovery of drugs and the treatment of diseases [10, 11]. The application of bioinformatics text mining to drug data sets helps to improve the understanding of gene-disease-drug relationships [12–14]. For the past years, the successful introduction and implementation of many multicenter genomic studies, such as the use of micro-array, proteomics, and other high-throughput screening assays, provided the hundreds to thousands of interesting gene hits, which may present as different integration and phenotypes [15–18]. In the research of Aliyu [19] et al., the latest progress of Connectivity Map and the related methods of drug discovery are analyzed. It is proposed that Connectivity Map be used as an effective tool to study the characteristics of related drugs or diseases. Lei Xie [20] et al. pointed out that drug action is a complex process. Systematic pharmacology models based on genome-wide, heterogeneous, and dynamic data integration have shown promise in drug reuse [21, 22], prediction of drug side effects [23–26], development of combination therapies, and precise drugs [27]. However, these methods also have some shortcomings. Therefore, in order to better study the relationship between traditional Chinese medicine and disease, in this study, a possible solution to this analytical hurdle in the data mining may be found in a web-based approach. It is a simple but very effective method that has found application in the analysis of large amount of data acquired from different studies on many human diseases. This approach has been used to explore the relationships between the drug and its targets or the interacting protein related to that disease [28]. Bioinformatics Analysis Tool for Molecular mechANism of Traditional Chinese Medicine (BATMAN-TCM) is a web-based tool that allows scholar-researchers to get the information about various drug targets and the related diseases.

In our study, we used BATMAN-TCM to find out the information about the multiple active ingredients of the* Damnacanthus indicus C.F.Gaertn* and the corresponding 62 drug targets. We got 100 related proteins on string website through the protein-protein interaction analysis. After further analyzing the function of readout targets and their related genes through pathway enrichment analysis, the top 10 KEGG pathways were taken into consideration. One of the most significant cancer-related pathways is the breast cancer pathway. The Rap1 pathway was identified as one of the top 10 enriched KEGG pathways which contain 14 genes related to* Damnacanthus indicus C.F.Gaertn* targets.

The medicine network pharmacology analysis is a new paradigm for the analysis of* Damnacanthus indicus C.F.Gaertn* and Rap1 interactions. The genes involved in* Damnacanthus indicus C.F.Gaertn, *i.e., Rap1, may be experimentally demonstrated and may elucidate the implications of* Damnacanthus indicus C.F.Gaertn* activation and provide new insights into the biological context of restoration of Rap1 function. This method can be useful to understand the beneficial effects of* Damnacanthus indicus C.F.Gaertn* in prevention of cancers and can help treat malignant tumors harboring the dysfunctional Rap1 signaling pathway.

## 2. Materials and Methods

The Minimum Standards of Reporting Checklist contains details of the experimental design and statistics and resources used in this study.

### 2.1. Drug-Target Search

The Bioinformatics Analysis Tool for Molecular Mechanism of TCM (BATMAN-TCM: http://bionet.ncpsb.org/batman-tcm/) is a very useful tool to analysis the traditional Chinese medicine and its functions. It can perform following function: (a) TCM ingredients' target prediction; (b) functional enrichment analysis of the targets, including biological pathway, and gene ontology functional terms and diseases; (c) the visualization of ingredient-target-pathway/disease association network and KEGG/Bicarta pathway with chosen targets; (d) and comparison analysis of multiple traditional Chinese medicines [29]. In this study, we used this tool to search for the active ingredients of* Damnacanthus indicus C.F.Gaertn* and their targets.

### 2.2. Web Generation/Visualization and Genome Enrichment Analysis

Through the use of string search and functional protein association network function, drug protein and second level protein-protein interactions data were first generated for* Damnacanthus indicus C.F.Gaertn*. WebGestalt (http://www.webgestalt.org/option.php), a comprehensive web-based data mining tool, was used to analyze the bioinformatics and attribution embedded in the gene sets [30, 31]. Enrichment analytic tools through the KEGG approach were used in WebGestalt to get the biochemical pathways and functions associated with this PPI network. The tissue-verified significant gene sets were then organized based on the KEGG pathways in a KEGG Table. Out of these, the top 10 pathways with P-value less than 0.01 were chosen.

### 2.3. Exploring the Cancer Genomics Data Related to* Damnacanthus Indicus C.F.Gaertn* through cBio Portal

The cBio Cancer Genomics Portal (http://www.cbioportal.org/) is an open access platform for encapsulating the molecular profiling data obtained from the cancer tissues and the cell lines into an easy-to-understand genetic, epigenetic, and gene expression data to explore the multidimensional cancer genomics data [32]. The portal's query interface provides an easy access to complex cancer genomics data and makes exploring and comparing the genetic changes easily understandable to researchers. The basic data thus obtained can be correlated with the clinical outcomes to promote new findings in the biological systems. In this study, the cBio portal was used to explore the possible relationship of* Damnacanthus indicus C.F.Gaertn*-related genes with the breast cancer in all the studies in this database.

By using the portal's search function,* Damnacanthus indicus C.F.Gaertn*-related genes in all samples of the breast cancer studies were classified as changed or unchanged. The OncoPrint was utilized as a heat map to provide genomic data sets—a visually appealing display of changes in the gene arrays in various tumor samples [33]. Another feature of the portal is that it can generate multiple visualization platforms by grouping prostate cancer data alterations using input from gene sets [33–36].

### 2.4. Survival Analysis by UALCAN

UALCAN (http://ualcan.path.uab.edu/index.html) is an interactive web resource for analyzing the cancer transcriptome data. UALCAN is designed to (a) provide a publicly available platform for cancer transcriptome data; (b) allow researchers to identify biomarkers or to perform in silico validation of the potential genes; (c) provide publication quality graphs and plots depicting gene expression and patient survival information based on the gene expression; (d) evaluate gene expression in the molecular subtypes; and (e) provide additional information about the selected genes using links to other databases such as HPRD, GeneCards, Pubmed, TargetScan, and the human Protein Atlas [37]. In our study, UALCAN was used to analyze the prognostic survival of three genes that replicate the Rap1 pathway to targets of the* Damnacanthus indicus C.F.Gaertn*.

## 3. Results

### 3.1. Characterization of the Active Constituents of* Damnacanthus Indicus C.F.Gaertn* Using BATMAN and Visualizes of* Damnacanthus Indicus C.F.Gaertn* Linkage Networks by Cytoscape

In the biological system, chemicals (such as drugs or chemopreventive agents) interact with proteins and genes, leading to physiological functions at the cell or organ level. This higher-order, complex interaction among molecules can be considered as a network that is usually composed of nodes (e.g., genes, proteins, and diseases) that represent biological entities and edges that represent the relationship among them. First, we searched BATMAN-TCM using* Damnacanthus indicus C.F.Gaertn* as input. The 16 main compounds of the* Damnacanthus indicus C.F.Gaertn* were found, four of which had no detailed chemical structure and none of the targets were predicted by these four compounds. The remaining 12 compounds had 62 primary direct protein targets (DPTs) (Table 1). Using “Protein-Protein Interaction (PPI)” in the string to expand our search and analysis, we got a total of 100 secondary DPT-associated proteins.

### 3.2. WebGestalt Was Used to Analyze the Functional Properties Associated with* Damnacanthus Indicus C.F.Gaertn*-Mediated Genomic Changes

**Figure 1:**
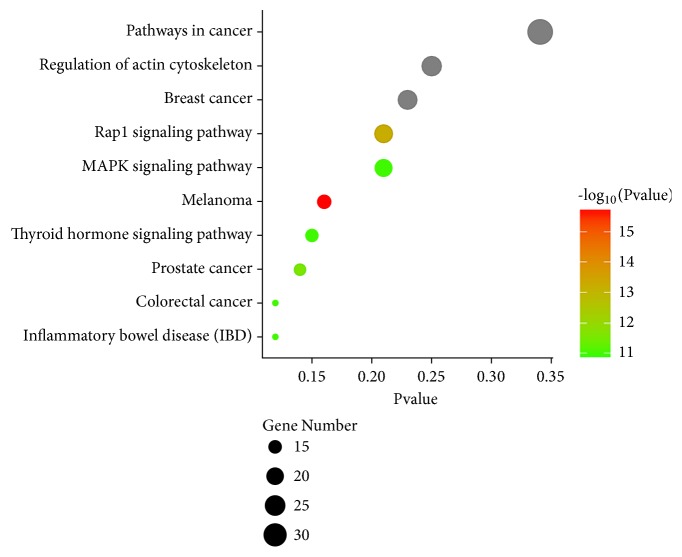
Plot of enriched gene sets identified using KEGG PATHWAYS ANALYSIS by Go Enrichment Plot tool.

To assess the functional characteristics of the* Damnacanthus indicus C.F.Gaertn*-mediated genome, we conducted the KEGG pathway enrichment analysis in WebGestalt. The top 10 KEGG pathways associated with DPT and DPT-related genes were chosen, which includes regulation of actin cytoskeleton (25 genes), pathways in cancer (34 genes), breast cancer (23 genes), Melanoma (16 genes), Rap1 signaling pathway (21 genes), MAPK signaling pathway(21 genes), prostate cancer (14 genes), colorectal cancer (12 genes), thyroid hormone signaling pathway (15 genes), and inflammatory bowel disease (IBD) (12 genes) (Table 2). Then, we used GO Enrichment Plot, which is one of tools on E chart (http://www.ehbio.com/ImageGP/index.php/Home/Index/), to visualize the pathway enrichment (Figure 1).

All of the enrichment pathways identified using this method represented a biological area that showed statistically significant correlation with* Damnacanthus indicus C.F.Gaertn* genomes and required further study. A broad panel of functional analysis indicated that* Damnacanthus indicus C.F.Gaertn*-related genes are predominantly cancer-associated and may have a functional association with the Raf1 pathway, thereby, suggesting the relationship of the* Damnacanthus indicus C.F.Gaertn* DPT with the Rap1 pathway. The enriched KEGG pathway derived from* Damnacanthus indicus C.F.Gaertn*'s genome also found 14 genes that overlap with the genes which are found in Rap1 pathway.

### 3.3. Acquisition of Genetic Alterations Associated with* Damnacanthus Indicus C.F.Gaertn* Related Genes MAP2K1, PIK3CA, and Raf1 by cBio Portal

**Figure 2:**
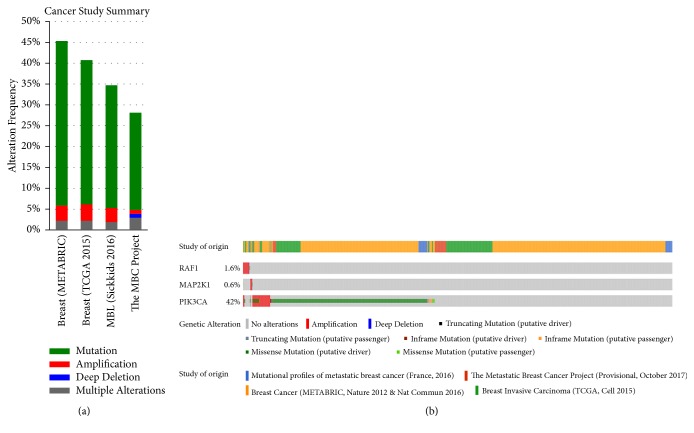
Mining genetic alterations connected with MAP2K1, PIK3CA, and RAF1, in breast cancer studies embedded in cBio cancer genomics portal.

Functional enrichment analysis showed a link between* Damnacanthus indicus C.F.Gaertn*-related genes and cancer-related pathways. We used the cBio portal to find out the genetic changes associated with the targets of the* Damnacanthus indicus C.F.Gaertn* in the breast cancer to prove the validity of this link. The Rap1 signaling pathway is the main target of the* Damnacanthus indicus C.F.Gaertn*, and we found three overlapping genes (MAP2K1, PIK3CA, and Raf1) in the Rap1 signaling pathway associated with the breast cancer. Four breast cancer studies analyzed the alterations of gene sets and presented for analysis [38–40] which ranged from 28.16% to 45.39% (Figure 2(a)). Using OncoPrint tool, a summary of the polygenic changes showing the most significant genomic changes in each group of tumor samples was made. The results showed that at least one of the three genes inquired changed in 1240 cases (43%). The frequency of change of each selected gene is shown in Figure 2(b). For RAF1, most of the alterations are amplification mutations and a few are deep deletions, truncated, and missense mutations. Gene changes associated with MAP2K1 include deep deletions and amplification and a few missense or truncation mutations. For PIC3KCA, the majority of mutations are amplification mutations, along with a small amount of inframe and truncation mutations and missense mutations.

### 3.4. Survival Analysis

**Figure 3:**
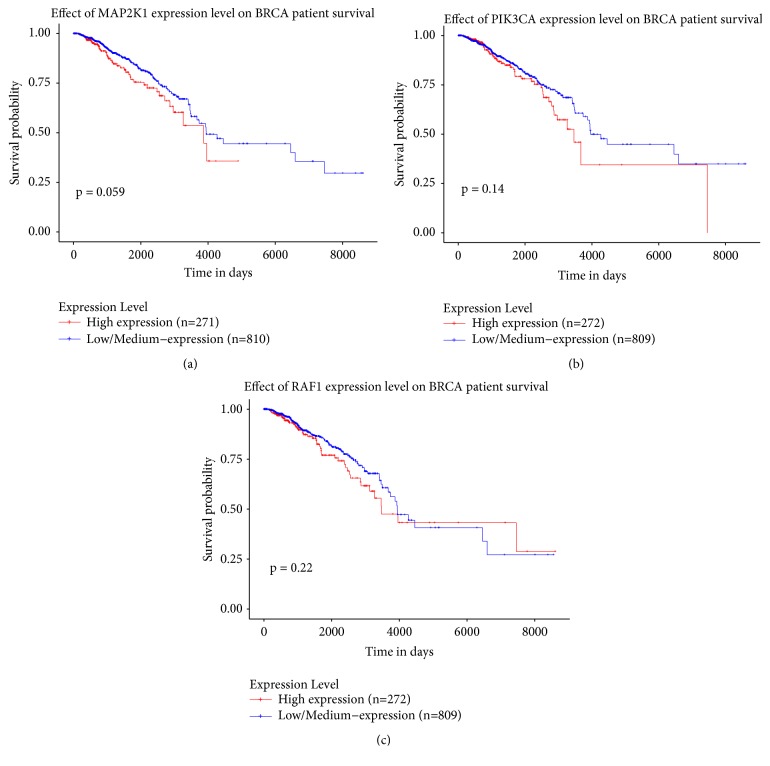
(a) Kaplan-Meier plot indicating effect of MAP2K1 expression level on BRCA patient survival. (b) Kaplan-Meier plot indicating effect of PIC3CA expression level on BRCA patient survival. (c) Kaplan-Meier plot indicating effect of RAF1 expression level on BRCA patient survival.

For the survival analysis using UALCAN as a tool, three genes of MAP2K1 (Figure 3(a)), PIK3CA (Figure 3(b)), and Raf1 (Figure 3(c)) were used the input, and TCGA dataset was chosen. Breast invasive carcinoma was selected as the data set to obtain the survival curves of three genes in the breast cancer patients. Output analysis showed that, in the breast cancer patients, MAP2K1 and PIK3CA have a significant relationship with the occurrence and prognosis of the breast cancer.

## 4. Discussion

The wide range of beneficial effects of* Damnacanthus indicus C.F.Gaertn* is still underexplored and underreported. Therefore, there is a need for new analytical methods or platforms that can link the* Damnacanthus indicus C.F.Gaertn* to its target protein so as to prove its relationships with the observed biological effects. In this study, we explored the TCM mechanism based on the molecular network through the research route of TCM pharmacology into four steps: (1) identify the effective active ingredients of the TCM; (2) identify the genes related to the diseases treated by TCM and construct the disease network; (3) determine the signaling pathways and networks controlled by the TCM targets and evaluate the influence of the TCM on the disease networks; (4) study the related genes for the survival analysis. We have conducted this study using a set of web-based tools to elucidate the molecular mechanisms of* Damnacanthus indicus C.F.Gaertn* and their relationship with the clinical outcome of cancer.

In this study, we found 62 major DPTs, 100 secondary DPT-related genes/proteins, and 10 KEGG pathways rich in genes related to* Damnacanthus indicus C.F.Gaertn*. Based on the functional characteristics of the known 100 DPT-related genes and our current understanding of the KEGG pathway, 31 genes may be considered as the secondary targets of* Damnacanthus indicus C.F.Gaertn*'s bone associated with cancers in the human body. The genetic alterations in the three overlapping genes (MAP2K1, PIK3CA and RAF1) revealed by the* Damnacanthus indicus C.F.Gaertn* prick-related rap1 signal further explored and assessed for the beneficial effects which* Damnacanthus indicus C.F.Gaertn* may exerts in the treatment of various cancers. The MAP2K1, PIK3CA, and RAF1 and especially Raf1 are all believed to be the common suppressors in the breast cancer [41].

Through the molecular network analysis, our study shows the association of anticancer effects of the* Damnacanthus indicus C.F.Gaertn* with its main target proteins as well as the* Damnacanthus indicus C.F.Gaertn*-related genes. In this analysis, a functional link was found to be present between the pathways related to* Damnacanthus indicus C.F.Gaertn* and the effects observed in diseases such as breast cancer. Although we have shown that a link exists between* Damnacanthus indicus C.F.Gaertn* and breast cancer, this work can be extended to study the use in other solid tumors as well. In principle, the* Damnacanthus indicus C.F.Gaertn* link network revealed by the BATMAN-TCM tool can be applied to the diseases that are known to be affected by* Damnacanthus indicus C.F.Gaertn*.

A single TCM contains multiple chemical components, so the same efficacy can be achieved at much lower effective doses than a normal single chemical component. The characteristics of these drugs, such as requirement of low dose, multiple targets, and less chances of development of the resistance and small side or toxic effects, make the TCM unique in the treatment of certain complex diseases regulated by multiple genes and chronic diseases requiring long-term medication. The concept of “multi-target drug” based on the molecular networks is gradually becoming a new trend [42] and has become an academic hotspot in biology, pharmacology, chemistry, and medicine for the resources and inspiration trend [43].

There are lots of indexes to evaluate the effects of these subcombinations. Using a systematic biology research to build a network of pharmacological effects, such as using the DrugBank database [44], HIT (Herbal Ingredients' Targets Database) [45], Drug-Targets Library (drug-target network) [46], human disease library [47], and other resources can reveal the biocompatibility of the compound and their complex molecular mechanisms. In a study by Fang [48], they found out the antirheumatoid Huanglian Jiedu decoction mechanism by searching on HIT, DrugBank database for the targets information. At the same time, they also extracted corresponding 32 FDA approved medicines and found five corresponding targets in the Huanglian Jiedu Tang. The target proteins of the TCM were consistent with the target proteins of the three western medicines, which explain the compounds' antirheumatoid effect to a certain extent.

For the past years, the study of TCM network pharmacology proves that studying the main components of the TCM and its target role in the context of cellular networks can help us to understand the whole dialectical and coordinated views of TCM. We believe that elucidating the mechanism of action of TCM at the level of regulation of molecular networks will provide us the useful inspiration and evidence for the discovery of multitargeted drugs. It may also reverse the development of modern multicomponent new drugs from clinically effective TCM and modernization of TCM will also play a positive role in promoting these medicines.

TCM network pharmacology research is still in the initial stage of the development, and it needs continued research to develop the new potential and application prospects. However to achieve these goals, pharmacology department is lagging behind, keeping in view the current status of the systemic pharmacology and technology development. Our outlook is as follows:

(1) For already developed drug combinations, there is a need to develop more systematic approaches to understand the mechanism of action of drug combinations. Learning about the differences and similarities of different drug combinations provides the theoretical and practical basis for the combination therapy of complex diseases.

(2) Further exploration and optimization of the web analytics, as well as a complete drug discovery network, are needed. Currently, the accuracy of the network is limited due to the numerous pitfalls and these are lacking in providing sufficiently high-quality data. Also, there are still many other factors such as environmental stress, epigenetic modifications, and intrusion of pathogen, information of which cannot be integrated into the network.

(3) There is a felt need for the more systematic methods to solve the complexity of questions asked on TCM, to figure out the basic theory of TCM and to provide a comprehensive and systematic theoretical guidance on the multicomponent multitarget characteristics of TCM.

(4) Reasonable experiments are needed to verify the feasibility of the results. We can take advantage of the great complexity of Library of Integrated Network-based Cellular Signatures (LINCS) data to provide guidance for the experimental design of the follow-up study [49].

## 5. Conclusion

This study provides a totally new and scientific way to holistically decipher that the pharmacological mechanisms of Damnacanthus indicus C.F.Gaertn in the treatment of breast cancer might be associated with MAP2K1, PIK3CA, and Raf1 genes. However, this study was only performed on the silicon which based on data analysis, and further experimental experiments were demanded to validate these hypotheses. In summary, the network pharmacology, which Tze-chen Hsieh et al. called functional/activity network (FAN) analyses [50], provides a flexible interface to test hypotheses in cancer and other diseases by calling data in databases contributing to researchers in translating basic research into clinical applications.

## Figures and Tables

**Table 1:** Identification of direct targets of resveratrol using BATMAN-TCM Searched.

Compound	Predicted targets [Gene Symbol] ranked according to the decreasing (score)
Damncanthal	This compound doesn't have any potential target with score larger than 20.
Nordamnacanthal	ESR1(23.00)
7-Hydroxyaloin	This compound doesn't have any potential target with score larger than 20.
18-Nordehydroabietan-4alpha-Ol	S1PR5(48.000)ESR2(48.000)ESR1(48.000)NR1I2(48.000)
Nor-Ketoagarofuran	AR(48.000)
2-Benzylxanthopurpurin	ESR1(80.882)HSD17B1(23.000)TFAP2C(22.373)TOX3(22.373)TGFB1(22.373)
	MED1(22.373)NKX3-1(22.373)ESR2(22.373)SHH(22.373)LEF1(22.373)
	FGFR2(22.373)PGR(22.373)WNT4(22.373)AREG(22.373)GPER1(22.373)
	WNT5A(22.373)SOX9(22.373)VDR(22.373)TRIM24(22.373)
Kadsudilactone	CHRM3(80.882)CHRM1(80.882)CHRM2(80.882)PGR(48.000)AR(48.000)
	NR3C2(48.000)ARFGEF2(22.373)DOCK5(22.373)NTSR1(22.373)CHRM5(22.373)
	GNA15(22.373)P2RX1(22.373)DOCK4(22.373)MAP2K1(22.373)CHRM4(22.373)
Damnacanthal	This compound doesn't have any potential target with score larger than 20.
Juzunal	This compound doesn't have any potential target with score larger than 20.
Digiferrugineol	CHRM3(23.000)CHRM1(23.000)CHRM2(23.000)CHRM4(23.000)
Damsin	PGR(80.882)AR(80.882)CYP19A1(80.882)NR3C2(80.882)WNT4(55.444)
	CACNA1I(23.000)CACNA1G(23.000)TNFSF11(22.373)COL5A1(22.373)NOX1(22.373)
	PHB(22.373)MED1(22.373)NKX3-1(22.373)ESR1(22.373)NEDD4(22.373)
	SHBG(22.373)SERPINB3(22.373)FOXP3(22.373)C5(22.373)SRC(22.373)
	STAP1(22.373)TGFB2(22.373)MMP28(22.373)UBR5(22.373)IL10(22.373)
	NR1I3(22.373)TNFAIP3(22.373)ALDH1A1(22.373)IL4(22.373)TSPO(22.373)
	CX3CR1(22.373)CD34(22.373)S100A9(22.373)PDGFB(22.373)VDR(22.373)
Norjuzunal	ESR1(23.000)

**Table 2:** List of enriched gene sets identified using KEGG PATHWAYS ANALYSIS.

Pathway Name	#Gene	OverlapGene	Statistics
Regulation of actin cytoskeleton	25	1128 1129 1131 1132 1133 1398 1950 2246 2247 2248 2249 2251 2252 2254 2255 2263 3265 5155 5290 5604 5829 5894 6714 673 9564	C=216; O=25; E=2.68; R=9.33; PValue=0e+00; FDR=0e+00

Pathways in cancer	34	1398 1499 1950 2246 2247 2248 2249 2251 2252 2254 2255 2263 2736 3265 354 367 4087 4088 4824 51176 5155 5290 54361 5604 5727 5894 595 64399 6469 673 6774 7040 7042 7046	C=397; O=34; E=4.92; R=6.9; PValue=0e+00; FDR=0e+00

Breast cancer	23	1499 1950 2099 2100 2246 2247 2248 2249 2251 2252 2254 2255 3265 51176 5241 5290 54361 5604 5894 595 673 8600 8648	C=146; O=23; E=1.81; R=12.7; PValue=0e+00; FDR=0e+00

Melanoma	16	1950 2246 2247 2248 2249 2251 2252 2254 2255 3265 5155 5290 5604 5894 595 673	C=71; O=16; E=0.88; R=18.17; PValue=2.22e-16; FDR=1.68e-14

Rap1 signaling pathway	21	1398 1499 1950 2246 2247 2248 2249 2251 2252 2254 2255 2263 3265 5155 5290 5604 5894 6714 673 9564 9732	C=212; O=21; E=2.63; R=7.99; PValue=6.31e-14; FDR=3.82e-12

MAPK signaling pathway	21	1398 1950 2246 2247 2248 2249 2251 2252 2254 2255 2263 3265 5155 5604 5894 673 7040 7042 7046 8911 8913	C=255; O=21; E=3.16; R=6.64; PValue=2.43e-12; FDR=1.11e-10

Prostate cancer	14	1499 1950 2263 3265 354 367 4824 51176 5155 5290 5604 5894 595 673	C=89; O=14; E=1.1; R=12.68; PValue=2.56e-12; FDR=1.11e-10

Colorectal cancer	12	1499 4087 4088 51176 5290 5604 5894 595 673 7040 7042 7046	C=62; O=12; E=0.77; R=15.6; PValue=8.08e-12; FDR=3.06e-10

Thyroid hormone signaling pathway	15	10499 1499 2099 3265 5290 54361 5469 5604 5894 595 6714 8648 9440 9611 9862	C=118; O=15; E=1.46; R=10.25; PValue=9.73e-12; FDR=3.27e-10

Inflammatory bowel disease (IBD)	12	3558 3561 3565 3566 3586 4087 4088 50943 6774 6778 7040 7042	C=65; O=12; E=0.81; R=14.88; PValue=1.46e-11; FDR=4.43e-10

## Data Availability

The data used to support the findings of this study are available from the corresponding author upon request.
